# Overcoming Template Surface Blocking: Geraniol Adsorption Studies Guiding MIP-Based Sensor Design

**DOI:** 10.3390/ijms262311454

**Published:** 2025-11-26

**Authors:** Greta Kaspute, Deivis Plausinaitis, Vilma Ratautaite, Evelina Vaicekauskaite, Vytautas Bucinskas, Arunas Ramanavicius, Urte Prentice

**Affiliations:** 1 Department of Nanotechnology, SRI Center for Physical and Technological Sciences (FTMC), Sauletekio Av. 3, LT-10257 Vilnius, Lithuania; greta.kaspute@ftmc.lt (G.K.); vilma.ratautaite@ftmc.lt (V.R.); arunas.ramanavicius@chf.vu.lt (A.R.); 2 Department of Personalised Medicine, Innovative Medicine Center (IMC), Santariskiu Str. 5, LT-08406 Vilnius, Lithuania; 3 Department of Physical Chemistry, Institute of Chemistry, Faculty of Chemistry and Geosciences, Vilnius University (VU), Naugarduko St. 24, LT-03225 Vilnius, Lithuania; deivis.plausinaitis@chf.vu.lt (D.P.); evelina.vaicekauskaite@chgf.stud.vu.lt (E.V.); 4 Department of Mechatronics, Robotics and Digital Manufacturing, Faculty of Mechanics, Vilnius Gediminas Technical University, Plytines St. 25, LT-10105 Vilnius, Lithuania; vytautas.bucinskas@vilniustech.lt

**Keywords:** geraniol adsorption, pyrrole adsorption, molecularly imprinted polymers, competitive adsorption, selective adsorption

## Abstract

To develop molecularly imprinted polymer (MIP)-based biosensors effectively, it is necessary to evaluate the potential adsorption of materials onto the electrode surface. Therefore, we investigated the adsorption of geraniol and pyrrole and compared them. In addition to determining adsorption constants, particular focus was placed on adsorption mechanisms, as they directly influence monolayer or multilayer formation, template removal efficiency, and the selectivity of the final imprinted structure. To achieve this, we employed various electrochemical methods, including cyclic voltammetry (CV), electrochemical impedance spectroscopy (EIS), and quartz crystal microbalance (QCM) measurements. Measurements were repeated to ensure reliability. The findings were used to calculate adsorption constants using the Langmuir equation. Geraniol and pyrrole showed adsorption constants of 21.5 L/mol and 31.7 L/mol, respectively, indicating strong molecular interactions. These results indicate strong interactions between the two molecules, suggesting that geraniol influences electrode polymerization. This led to the importance of proper surface preparation, evaluation of analyte–monomer interactions, and the opportunity to reuse materials.

## 1. Introduction

Geraniol is an acyclic monoterpenoid alcohol, which has the formula C_10_H_18_O [1]. It is valued for its sweet, floral, rose-like scent, and can be found in various cosmetic products [2]. Despite its odor, this essential oil (EO) component has repellent and insecticidal properties with low mammalian toxicity [1]. Additionally, creams containing 3% geraniol have been shown to inhibit the growth of *E. coli*, *C. albicans*, and *T. rubrum*. However, it has limited effects on certain mold fungi [3]. Nevertheless, geraniol also exhibits biological activities, including antitumor [4,5], anti-inflammatory [6,7], antioxidant [8,9], hepatoprotective [10,11], cardioprotective [12,13], and neuroprotective properties [14,15]. From a chemical perspective, it is a lipophilic compound, which is notable for its enhancement of transdermal drug delivery. Encapsulation of geraniol improves its dispersion in aqueous systems, which is important for producing an effective drug or food preservative [7,16].

Adulteration can be detected in many products, such as milk [17,18], honey [19], vanilla [20,21], etc. In food adulteration cases, unregulated substances are added, or valuable ingredients are substituted with cheaper alternatives. This results in product quality reduction. In the EOs adulteration case, less expensive oils are blended with pure ones, or synthetic compounds are added [22]. Reliable detection methods are needed to ensure products are safe, non-toxic, and naturally derived. Although geraniol adulteration is a growing concern, its selective recognition still lacks sufficient fundamental data and developed detection tools. As a preliminary step toward future detection strategies, the adsorption behavior of geraniol must be well understood to design selective analytical approaches. Therefore, this study does not focus on developing a complete sensor but instead aims to understand geraniol behavior on electrode surfaces prior to imprinting, which is essential for forming stable and selective host–guest interactions.

The adsorption process plays a critical role in molecular imprinting, as it determines whether the analyte is effectively oriented and retained during polymer formation. Adsorption can generally occur through physical forces (physisorption) or chemical bonding (chemisorption), which influences both the stability of the formed cavities and the ease of template removal [23]. To evaluate these interactions, adsorption models such as the Langmuir and Freundlich isotherms are commonly applied [24,25]. The Langmuir model assumes monolayer adsorption onto a homogeneous surface, which is desirable for imprinting [24], while the Freundlich model describes heterogeneous multilayer adsorption. The Freundlich model may hinder complete analyte extraction and reduce binding site specificity [26]. Therefore, analyzing equilibrium adsorption parameters and surface coverage provides essential information on the suitability of the chosen monomer–template–electrode system for generating selective recognition sites.

Molecularly imprinted polymers (MIPs) can serve as a tool to address the problem of adulteration. MIPs are synthetic materials that are developed with specific binding sites to target analytes in complex mixtures. To develop effective MIP design, understanding the initial adsorption interaction between template, monomer, and electrode is crucial. Molecular imprinting technology enables the development of durable recognition materials, where a polymer network is formed around an analyte molecule, and the analyte is subsequently removed, leaving selective binding sites. These sites further interact with the target molecule through hydrogen bonding, dipole–dipole interactions, and ionic forces [27,28]. MIPs combine the specificity and selectivity of biological receptors with advantages such as environmental durability and cost-effectiveness [28,29,30,31]. MIPs can serve as a selective adsorption system even in very low concentrations of target analytes [32].

MIP-based sensors can be developed for chromatographic applications or be electrochemical ones. In chromatography, MIPs serve as sorbents in solid-phase extraction, or high-performance liquid chromatography (HPLC). This method serves high selectivity even at low analyte concentrations. The mechanism is that in normal-phase mode, analytes are selectively retained through shape-specific interactions, while interfering molecules are simultaneously removed. In reverse phase modes, hydrophobic interactions retain analytes from aqueous solutions. Its selective binding is maintained through the washing process [33]. However, despite many advantages, chromatographic techniques face limitations such as the need for complex optimization of flow rates, mobile phase composition, and elution conditions, which can limit their suitability for rapid or on-site analysis. Chromatography provides excellent analyte separation and purification but often requires longer analysis times and complex instrumentation [33].

In the case of electrochemical MIP-based sensors, these systems offer a complementary solution to chromatographic limitations. The MIP layer is formed directly on the electrode, allowing for real-time analyte recognition using techniques such as cyclic voltammetry (CV), electrochemical impedance spectroscopy (EIS), or quartz crystal microbalance (QCM) [34,35]. These electrochemical methods are used to identify adsorption kinetics and polymerization. This is crucial for optimizing sensor selectivity and sensitivity. Electrochemical sensors enable rapid, cost-effective, and potentially portable detection, but achieving high selectivity and stability requires precise control of polymerization conditions and analyte–monomer interactions [36].

The selection of pyrrole as the monomer for the polymer layer was based on its well-established electrochemical polymerization characteristics and widespread use in conducting polymer research. However, the combined adsorption behavior of pyrrole and geraniol in sensor development has not yet been analyzed [37,38,39]. Here, we address the gap between investigating analyte adsorption and its relevance to MIP preparation as a key step toward MIP-based biosensors, since understanding these interactions is crucial for maintaining electrode stability, promoting polymerization, and enhancing imprinting efficiency. Moreover, this study provides essential insights into adsorption required prior to constructing a functional MIP sensor, particularly regarding whether geraniol forms single- or multilayer adsorption and whether complete template removal is always achievable. These adsorption characteristics ensure that the generated cavities remain functional for rebinding and will guide further optimization and integration of the MIP material into a selective geraniol sensor.

## 2. Results and Discussion

### 2.1. CV Results with System I

The morphology of Ppy layers plays a crucial role in the performance of Ppy-based sensors. A major challenge in developing these sensors is producing Ppy layers with predictable morphology. The Ppy layer morphology is influenced by numerous synthesis parameters, including the composition and concentration of the polymerization solution, temperature, electrode material, and surface roughness, and the electrochemical conditions during polymerization [35]. Variations in any of these factors can significantly affect the structure of the resulting layers, which in turn influences their adsorption behavior and the formation of defects or delamination, potentially affecting the performance of MIPs integrated with the sensor.

The operating principle of electrochemical sensors relies on recording electrical signals, such as current, charge, or potential, generated at the electrode surface in response to the target analyte [40]. For potential cycling, critical parameters include the number of cycles, sweep rate, and potential range, while constant-potential deposition depends primarily on the applied potential and its duration. In electrochemical MIP deposition, monomer and template concentrations, solvent composition, and pH are important, although this method typically requires less optimization than chemical polymerization. The latter involves additional steps to immobilize MIPs on the electrode, with factors such as spin-coating, dipping, or suspension deposition influencing the quality of the coating. Template extraction is often efficiently achieved by simple water washing at elevated temperatures (~80 °C) [41].

In this study, particular attention was required due to the use of geraniol, a hydrophobic compound with low water solubility, which poses challenges for the QCM system and may cause blockages or inconsistent mass readings. Its chemical properties, including limited solubility and tendency to form aggregates, could lead to uneven polymer layer formation or partial adsorption on the electrode surface. Therefore, classical electrochemical polymerization on the electrode (system I) was essential for monitoring and controlling the layer formation under these conditions.

**Figure 1:**
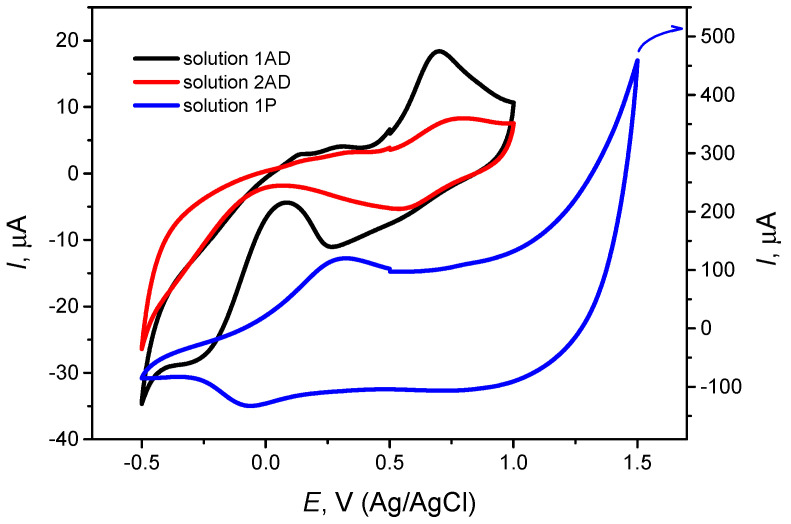
Comparison of CV responses in 1AD, 2AD and 1P solutions. Measurements of 1AD and 2AD were performed within the same potential range of −500 mV to +1000 mV vs. Ag/AgCl, with 3 cycles at a scan rate of 100 mV/s. The 1P measurement was carried out separately under similar conditions (−500 mV to +1000 mV vs. Ag/AgCl, 5 cycles, scan rate 100 mV/s). The second cycle of each CV is represented in the figure.

The oxidation and reduction potentials of pyrrole and geraniol were measured by CV. To achieve this goal, CV measurements were conducted using a 1P polymerization solution. Parameters used for CV were −500 mV to +1000 mV, repeated 5 times, max current 0.2 mA. These parameters were used because the Ppy layer developed efficiently, and the resulting layer had the expected brownish color. Electrodeposition of Ppy begins at almost similar potentials, and the level changes of the graph in each cycle show Ppy layer formation (Figure 1). In comparison, Marandi et al. evaluated the adsorption of pyrrole and the subsequent electropolymerization processes onto partially atomically flat Au (111) surfaces from an aqueous solution containing 0.1 M pyrrole and 0.1 M LiClO_4_ using non-contact atomic force microscopy [42]. Electrodeposition of Ppy begins at approximately the same potentials. However, the charge consumed in the same potential range (from 0.0 mV to +750 mV) is significantly lower for the case of the adsorbed pyrrole layer—more than 10 times less—compared to the electropolymerization in the bulk solution. This suggests that the adsorbed pyrrole layer undergoes a more efficient or less extensive polymerization process under the same conditions [42]. In our research, we conducted a thin Ppy-layer peroxidation process, as illustrated in Figure 1, to stabilize the electrode surface. This step was necessary because the experiments were performed in organic solutions rather than aqueous media, where surface stability is critical for reliable adsorption and polymerization behavior. According to the graph in Figure 1, we can identify the oxidation process starting from +250 to +1400 mV, and the reduction potential from −100 mV to −500 mV. Per oxidation starts in the range of +1100 to +1400 mV for a 1P solution. As the peroxidation is explained in previous studies, large nuclei are initially reduced in size, resulting in honeycomb-like patterns interspersed with ensembles of medium-sized nuclei. These structural transformations highlight the dynamic nature of the electropolymerization process, in which the electrode surface is reorganized into more ordered and finer-scale features [42].

To compare the adsorption behavior of pyrrole and geraniol solutions, CV was performed using a reference solution (1AD). The results were compared with those obtained for the geraniol-containing solution (2AD). The CV oxidation potential of 1AD was +600 mV to +900 mV, and the reduction potential was +200 mV to −250 mV. Comparing solution 2AD shows that oxidation and reduction potentials are significantly less expressed, resulting in oxidation at +550 mV to +600 mV and reduction at 250 mV to −100 mV. This means that geraniol, which is in the 2AD solution, blocks the surface of the electrode. Consequently, the formation of the MIP layer is strongly influenced by both the analyte and the polymer, as well as their respective adsorption capacities on the electrode surface.

### 2.2. Electrochemical Impedance Spectroscopy Results with Pyrrole and Geraniol Solutions

EIS parameters were evaluated for both pyrrole and geraniol solutions to gain insights into the interfacial properties of the gold electrode. EIS results are presented as the intersection of C_real_ and Z_real_, following the approach reported in previous studies by Plausinaitis et al. [35,40]. This representation allows for a clear visualization of changes in the electrode’s capacitive and resistive behavior during polymerization and facilitates fitting the data to an equivalent electrical circuit to evaluate whether the theoretical model adequately describes the system. In this context, ClO_4_^−^ is an inert anion, and Li^+^ is a cation, both of which exhibit minimal adsorption on the gold electrode surface. Therefore, LiClO_4_ serves as an ideal supporting electrolyte, enabling accurate assessment of changes in the capacitance of the target material over time.

**Figure 2:**
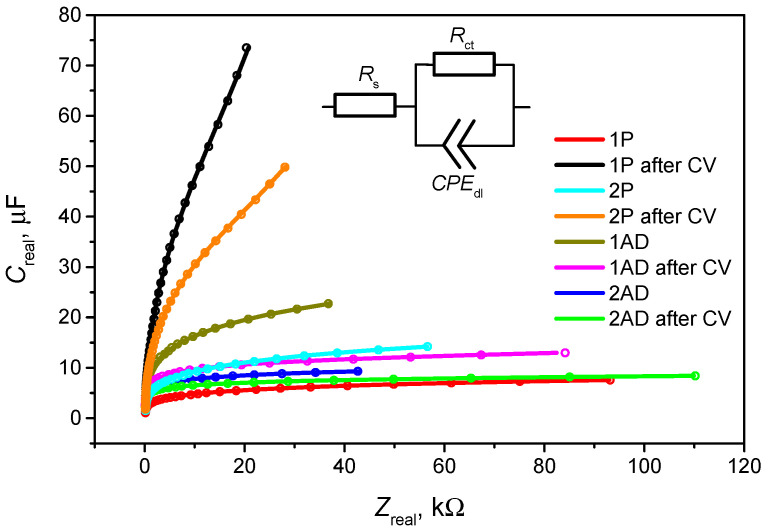
Comparison of EIS results of 1AD, 2AD, 1P, and 2P solutions before and after CV. EIS conditions: frequency range 10,000 Hz → 2 Hz, 10 points per decade. Inset: the Randles equivalent circuit. *R*_s_ is the resistance of the electrolyte, CPE is the constant phase element of electrical double layer, and *R*_ct_ represents the charge transfer resistance of Ppy layer in this equivalent circuit.

As shown in Figure 2, the data allow for the estimation of the double-layer capacitance, which reflects changes in surface coverage and adsorption behavior. The results indicate that pyrrole adsorption significantly alters the electrode interface, leading to higher capacitance values consistent with the formation of a polymer layer. This can be indicated by C_real_ change from 0 to −3.5 × 10^−5^ F. Notably, the pyrrole-containing 1P solution remained stable after CV, indicating sustained adsorption and polymerization. In contrast, geraniol produces smaller changes in capacitance, shifting from 0 to −5 × 10^−6^ F, suggesting weaker and more limited adsorption on the gold surface. These observations are consistent with the CV and QCM results, reinforcing the conclusion that pyrrole readily forms a stable layer suitable for molecular imprinting, whereas geraniol adsorption is slower and less extensive. The comparative EIS analysis confirms that the adsorption behavior of the monomer (pyrrole) and the analyte (geraniol) critically influences the formation and characteristics of the MIP layer.

**Figure 3:**
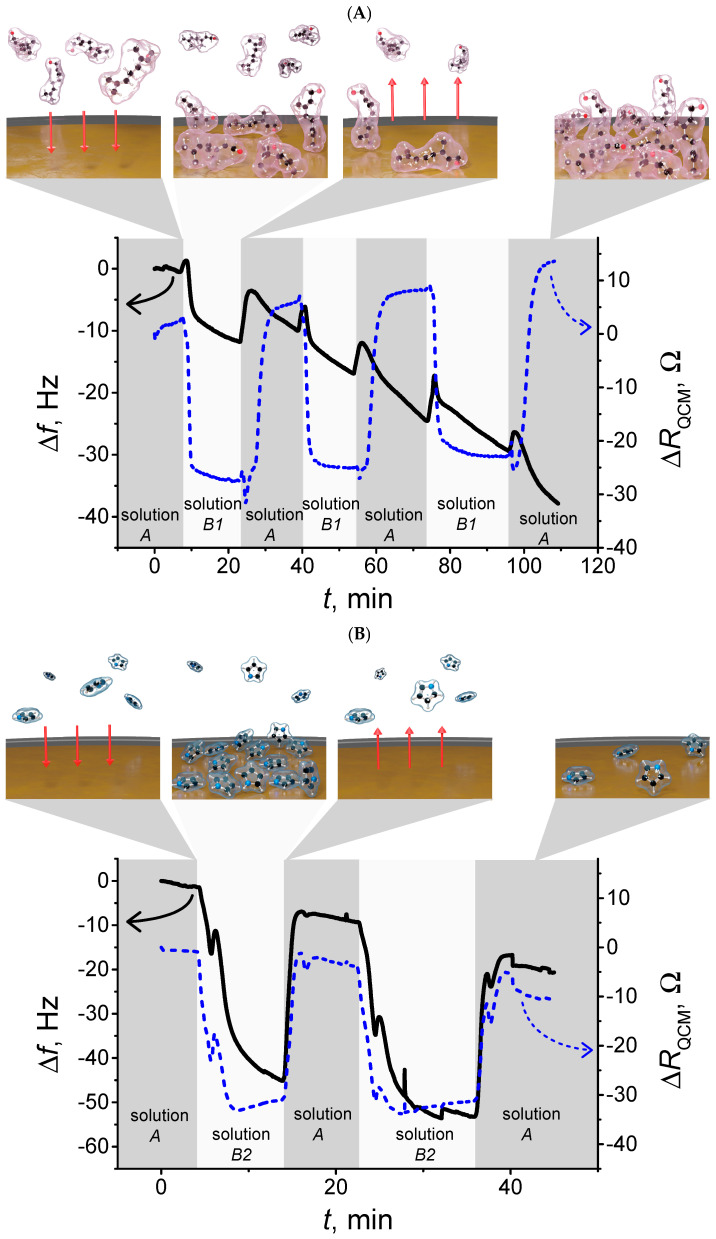
(**A**) Adsorption of 0.05 mol/L geraniol (solution B1) on the Au sensor, flow rate: 1 mL/min, applied twice, compared with solution A. (**B**) Adsorption of 0.5 mol/L pyrrole (solution B2) on the Au sensor, flow rate: 1 mL/min, applied twice, compared with solution A. Red arrows represent adsorption and then extraction.

Immediately after formation of the Ppy layer via electrochemical polymerization, EIS measurements were performed, and the recorded Z_real_-C_real_ spectra are shown in Figure 3. At low frequencies, the curves show a marked decrease in parallelism with the Z_real_ axis compared to measurements taken before pyrrole polymerization, indicating that the electrode surface has become more electrochemically heterogeneous [31]. The presence of geraniol exhibits a pronounced effect on the formation of the Ppy layer. Comparative analysis of the systems 1P/1P after CV and 2P/2P after CV demonstrates a clear distinction between the samples. In the pyrrole-containing solution, a significant increase in C_real_ is observed following CV measurements, confirming the effective formation of a Ppy layer. Conversely, in the pyrrole–geraniol solution, only a minor change in Creal is detected, indicating that the incorporation of geraniol suppresses Ppy growth and thereby limits the electrodeposition process.

After fitting the EIS data, the equivalent parameters obtained according to the Randles equivalent circuit are presented in Table 1. As we can see by comparing the *CPE*_dl_ results for 1P/1P after CV with 2P/2P after CV, after polymerization in pyrrole solution (1P) the double layer capacitance increases by approximately 6.7 times, while in the pyrrole-geraniol solution (2P) it increases only by 2.8 times. The smaller increase in double-layer capacitance indicates a smaller amount of Ppy formed on the electrode surface.

### 2.3. QCM Findings Comparing Pyrrole and Geraniol Adsorption on the Au Sensor

The QCM method was used to get additional details about the analyte—geraniol and monomer—pyrrole adsorption. After completing the experiment, the number of molecules adsorbed on the Au sensor surface is calculated. During the experiment, 3D printed cell form organic glass issues were encountered as it swelled during the process, preventing the continuation of the experiment. For this reason, instead of using pure acetonitrile, a mixture of acetonitrile and water was chosen.

According to QCM results (Figure 3), geraniol adsorbates on sensor surface without reverse reaction, since geraniol does not de-adsorb from the gold surface in cleaning phase according to geraniol frequency curve (Figure 3A). Since pyrrole has the same property, the comparison of the pyrrole and geraniol adsorption is implemented during the research.

The adsorption mechanism in MIP–based sensors is governed by specific recognition sites formed during the imprinting process. These sites are complementary in shape, size, and functional groups to the target analyte, enabling selective binding at the sensor surface. In the case of QCM analysis, adsorption is detected as a change in resonance frequency (Δ*f*), which is directly proportional to the mass change (Δ*m*) on the electrode surface. This relationship is described by the Sauerbrey Equation [43]:

(1) $$\Delta f=-\frac{2f_{0}^{2}}{A\sqrt{\rho_{q}\mu_{q}}}\Delta m$$

where *f*_0_ is the fundamental frequency of the crystal, *A* is the active electrode area, *ρ_q_* is the density of quartz, and *μ_q_* is the shear modulus of quartz. The equation implies that higher mass adsorption onto the electrode surface leads to a larger decrease in resonance frequency.

To evaluate adsorption quantitatively, one typically examines the equilibrium binding isotherms (e.g., Langmuir or Freundlich models), which describe how surface coverage depends on analyte concentration [23]. To better characterize the adsorption behavior of geraniol, the experimental data were fitted using both Langmuir and Freundlich isotherm models. The Langmuir isotherm assumes monolayer adsorption onto homogeneous and specific binding sites created by imprinting, reflecting the ideal behavior of an optimized MIP, and is described by the Equation:

$$\frac{1}{Q_{e}}=\frac{1}{Q_{max}K_{L}C_{e}}+\frac{1}{Q_{max}}$$

In contrast, the Freundlich model accounts for heterogeneous surfaces and multilayer adsorption, which are also relevant for polymer materials, and is expressed as:

$$Q_{e}=K_{F}C_{e}^{\frac{1}{n}}$$

The comparative analysis of both models provides deeper insight into the surface properties of the MIP and confirms the presence of selective recognition cavities responsible for geraniol adsorption. From these isotherms, important parameters such as the binding constant (*K*) and maximum adsorption capacity (*Q_max_*) can be determined and used to evaluate the imprinting efficiency [25].

During QCM testing, solution concentrations were adjusted in-line to identify the lowest concentrations producing reproducible signals. Representative equilibrium concentration–adsorption pairs for every intermediate step were not retained; consequently, only the final reliable points (0.05 M geraniol, 0.5 M pyrrole) are available for quantitative comparison. These final concentrations were selected because they produced QCM responses that exceeded the experimental noise and could be reproducibly measured. We acknowledge that full Langmuir and Freundlich isotherm fitting requires multiple independent equilibrium points per analyte; this limitation is discussed below and we propose targeted experiments to obtain robust isotherms in future work.

According to the QCM results (Figure 3), pyrrole adsorbs readily onto the gold sensor surface, as indicated by a change in frequency of −43 Hz. In this experiment, the concentration of pyrrole was ten times higher than that used in the geraniol adsorption experiment. The molar ratio of pyrrole to geraniol was selected as 10:1, based on widely used molecular imprinting design principles for small organic molecules. In pre-polymerization conditions, the template molecule must be surrounded by a sufficient number of monomer molecules to ensure strong and stable intermolecular interactions that later define the geometry of the recognition cavities. Previous studies have demonstrated that for low-molecular-weight templates, at least ten monomer units are typically required to form a compact oligomeric structure around the analyte, preventing template aggregation and improving cavity fidelity after template removal [44]. This arrangement allows the formation of a pyrrole oligomer “ring” that encapsulates the geraniol molecule, which is essential for achieving selective binding in the final MIP [45]. Therefore, the 10:1 molar ratio was chosen to ensure favorable adsorption interactions, efficient pre-polymerization complex formation, and to support future imprinting performance. In contrast, geraniol adsorption on the gold surface produced a smaller frequency change of 10 Hz, suggesting that it exhibits a weaker interaction with the electrode than pyrrole. These observations indicate that, while pyrrole readily forms a stable polymer layer, geraniol adsorption is more limited, highlighting the need to carefully consider analyte–monomer interactions during MIP design.

A long-interval geraniol adsorption experiment was conducted using QCM to assess adsorption kinetics over time. Over 30 min, geraniol adsorption resulted in changes in a 38 Hz change in frequency and 4 Ohm change in resistance. It is important to note that the concentration of geraniol in this experiment was 10 times lower than that of pyrrole, yet geraniol still exhibited measurable adsorption over longer periods. This underscores the slower kinetics and lower surface coverage of geraniol compared to pyrrole, which has implications for MIP formation: the polymer matrix must accommodate the analyte’s adsorption behavior to ensure effective imprinting and high sensor specificity. These results collectively demonstrate the importance of tuning monomer-to-analyte ratios and exposure times in the development of MIP-based sensors for EOs.

To present comparable QCM measurement data, Table 2 summarizes pyrrole and geraniol adsorption on a gold surface. The parameters recorded include the average resonance frequency change (Δ*f*, Hz), the average resistance change (Δ*R*, Ohm), and the duration of each measurement period (*t*, min). Measurements were taken for two types of solutions: solution A, serving as the blank/reference, and solution B, containing either pyrrole or geraniol. Each row of the table represents the average changes in frequency and resistance over the specified time interval, for example, *t* = 0–10 min. The raw data show that pyrrole adsorbs approximately 3.3 times more on the gold surface than geraniol. After normalizing adsorption to account for this concentration difference, geraniol demonstrates a higher adsorption efficiency per unit concentration. This highlights that, while pyrrole produces a larger absolute QCM response, geraniol is more effective at binding relative to the amount of material present. These results provide insight into the adsorption behavior of pyrrole and geraniol on gold surfaces. Normalizing for concentration is essential for fair comparison, showing that geraniol may be a more efficient molecule for applications requiring effective surface binding at lower concentrations, such as sensors, coatings, or molecular electronics.

The adsorption mass per unit area (Δ*m*) was calculated from the QCM frequency shifts using the Sauerbrey equation:

(2) $$\Delta m=-\frac{\Delta fA\sqrt{\rho_{q}\mu_{q}}}{2f_{0}^{2}}$$

where Δf is the frequency change (Hz), A is the active electrode area, $\rho_{q}$ is the density of quartz (2.648 g·cm^−3^), $\mu_{q}$ is the shear modulus of quartz (2.947 × 10^11^ g·cm^−1^·s^−2^), and $f_{0}$ is the fundamental resonance frequency of the QCM crystal. The value of $f_{0}$ is specific to each QCM chip and corresponds to the resonance frequency measured for the crystal in air (i.e., before any liquid or film contact). This parameter is obtained from the manufacturer’s calibration or directly from the frequency response of the bare crystal. For the present measurements, the working electrode area was *A* = 0.79 cm^2^.

Pyrrole (10× concentration) showed Δ*m* ≈ 7.6 ng/cm^2^, while geraniol (1× concentration) showed Δ*m* ≈ 1.77 ng/cm^2^; when normalized per concentration, adsorption becomes 0.76 ng/cm^2^ for pyrrole and 1.77 ng/cm^2^ for geraniol. This indicates that, mole-for-mole, geraniol exhibits relatively strong interaction with the gold surface, although its absolute adsorption is lower due to the smaller solution concentration. Using the Langmuir equation [24]:

(3) $$q_{e}=\frac{q_{max}KC_{e}}{1+KC_{e}}$$

where: *q*_e_ is equilibrium adsorption capacity, *q_max_*—maximum adsorption capacity, *K*—Langmuir adsorption constant, *C_e_*—equilibrium concentration of adsorbate in solution. The adsorption constants were estimated as *K*_pyrrole_ ≈ 31.7 L/mol and *K*_geraniol_ ≈ 21.5 L/mol, confirming that both molecules interact effectively with the electrode, but pyrrole forms a more substantial molecular layer due to its higher concentration.

Using the reliable QCM endpoints, geraniol (0.05 M) produced an average adsorbed mass of ≈2.40 ng/cm while pyrrole (0.5 M) produced ≈7.92 ng/cm. When normalized by concentration, geraniol exhibited higher adsorption efficiency per molar unit. Because only single concentrations per analyte are available, formal Langmuir/Freundlich parameter estimation is not possible from the present dataset. Nonetheless, combined evidence from QCM kinetics (progressive Δ*f* and Δ*R* over time) and EIS (CPE α values significantly <1 and pronounced *R*_ct_ changes) indicates a heterogeneous adsorption environment and a range of site affinities. Therefore, although a Langmuir description provides useful estimates of monolayer capacity, a Freundlich-type description is likely to better represent the true adsorption behavior on electropolymerized surfaces. This heterogeneity should be considered in MIP fabrication to ensure accessible and selective recognition cavities.

**Figure 4:**
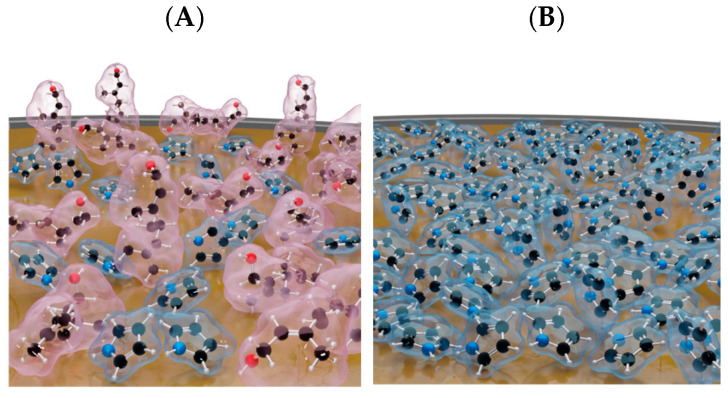
Gold surface models when the solution contains (**A**) pyrrole and geraniol together, i.e., 2P solution. (**B**) The solution contains only pyrrole. Red color represents geraniol molecules, blue—pyrrole.

By comparing the data obtained by EIS with QCM, we can make a preliminary conclusion that geraniol, being in a common solution with pyrrole, can also adsorb on the Au surface, i.e., compete with pyrrole. Thus, the surface concentration of pyrrole would be lower if we compare it with the result of a pure pyrrole solution (Figure 4). For this reason, the further electropolymerization of pyrrole to Ppy would proceed more slowly, because the adsorbed geraniol would interfere with it. Therefore, this knowledge is a useful result for creating MIPs with geraniol as the template molecule.

### 2.4. Limitations of the Study

First, the adsorption experiments were performed on bare gold surfaces rather than on completed MIP layers, and therefore represent a simplified model that does not fully replicate polymer–analyte interactions within imprinted cavities. Second, geraniol desorption was only evaluated with an acetonitrile–water system, and no systematic solvent optimization was conducted to ensure complete template removal. Third, only single concentrations of geraniol and pyrrole were analyzed for isotherm modeling, thereby preventing a more comprehensive evaluation of binding-site heterogeneity and competitive adsorption. Finally, this work did not proceed to fabrication and electrochemical testing of a fully imprinted polymer film, meaning that the sensor performance implications are based on indirect indicators rather than functional device testing.

These limitations will be addressed in future studies by implementing solvent optimization, expanding concentration ranges and template-to-monomer ratios, and fabricating multi-cycle polymer structures to validate the improved imprinting efficiency and analyte accessibility.

## 3. Experimental

### 3.1. Materials and Instrumentation

Chemicals: Pyrrole (98%, Alfa Aesar, Karlsruhe, Germany) was distilled before use for the preparation of MIPs. Acetonitrile (Carl Roth GmbH & Co., Karlsruhe, Germany) and geraniol (Sigma-Aldrich, Burlington, MA, USA) were used as received. Lithium perchlorate (LiClO_4_) (ThermoFisher, Erlangen, Germany) was used to prepare the side electrolyte. First-grade freshly distilled water (resistivity: 0.055 μS/cm at 25 °C) was used in all aqueous solutions. Ammonium hydroxide solution 25% (pure p.a. CAS No:1336-21-6, Poland), hydrogen peroxide ≥ 30% (p.a. CAS: 7722-84-1, Slovakia).

Electrochemical instrumentation: Electrochemical measurements were carried out using a Metrohm AutoLAB potentiostat/galvanostat (µAutolabIII/FRA2 µ3AUT71079, controlled by NOVA 2.1.3 software, EcoChemie, Utrecht, The Netherlands), and a Gamry Echem Analyst potentiostat/galvanostat (version 5.30, Warminster, PA, USA) [46]. For liquid handling and flow control, a binary HP 1100 HPLC pump (model G1312A, Hewlett-Packard, Germany) and a six-channel, two-position Rheodyne valve (Rohnert Park, CA, USA) were used to ensure constant, stable delivery. The aforementioned equipment was used to implement EIS [31] and chronoamperometry (CA) [32]. A 5 MHz electrochemical QCM (E-QCM) system (custom-built cell) was connected to an oscillator circuit and controlled by a Maxtek RQCM device (Cypress, CA, USA). Measurements were performed in real-time under flow conditions (0.5 mL/min).

#### Systems

Both electrode systems used in this study are presented in Table 3. Pictures of these systems–I and II—are presented in Appendix A.

**Figure 5:**
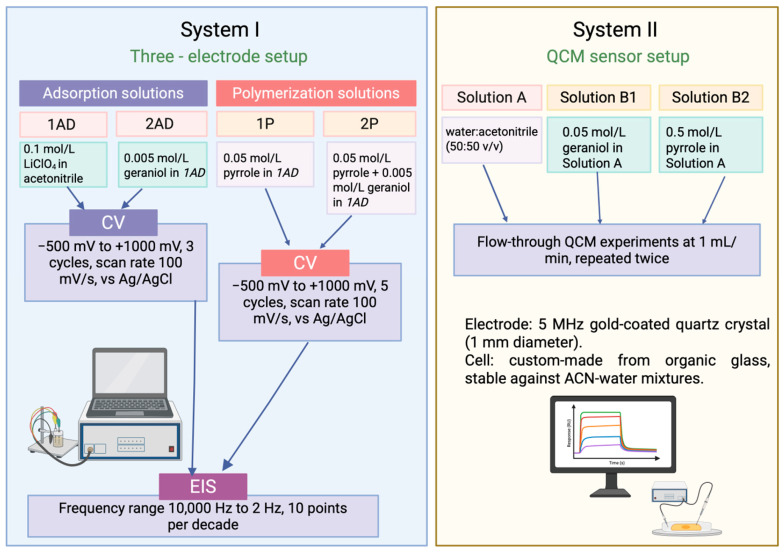
Schematic representation of system development and methods application. Created with BioRender.

The first system was applied to electrochemical experiments, including CV and EIS. These measurements were conducted to assess the interaction between the medium and the gold (Au) electrode, to determine suitable solutions and their optimal concentrations, and to establish the most suitable experimental conditions for this interaction. The second system was introduced once the potential ranges had been clearly defined using CV. At this stage, a QCM chip was employed, and QCM measurements were performed. To prevent the investigated material from completely dissolving in pure acetonitrile, water was added to the neutral solution. All measurements were repeated at least twice, and the experimental conditions are presented in Figure 5.

Overall, different solvent systems were used due to the requirements of each analytical technique. Electrochemical experiments required sufficient ionic conductivity. LiClO_4_ was added as a supporting electrolyte to enable charge transfer at the electrode interface. In QCM measurements, solution conductivity was not necessary, and to avoid introducing additional species that could affect the measured mass response, a solvent mixture of acetonitrile and water was used. This mixture ensured geraniol solubility while preventing potential chemical impact of pure organic solvent on the flow cell materials.

### 3.2. Pretreatment of the Working Electrode

The working electrode was pretreated for the electrochemical deposition of polypyrrole (Ppy) and geraniol according to established protocols [47,48]. Electrochemical cleaning was performed in a 0.1 M LiClO_4_ solution by cycling the potential between −500 mV and +1000 mV vs. Ag/AgCl at a sweep rate of 100 mV/s, repeated three times. CV confirmed the bare electrode surface and provided valuable insights into the electrochemical behavior of molecular species [49].

### 3.3. Au Sensor Regeneration

After experiments, the Au sensor was regenerated in piranha solution containing 60:40 (*v*/*v*) (25%) and hydrogen peroxide (≥30%). After experiments with solutions containing geraniol, the sensor was regenerated for 3 min, and after experiments with pyrrole for 5 min. All measurements were repeated at least 2 times.

## 4. Conclusions

Molecularly imprinted polymer (MIP) technology holds remarkable promise for monitoring food and cosmetic safety, as well as analytical applications; however, several obstacles still hinder its commercialization. Large-scale production remains limited by high synthesis costs, reproducibility issues, and challenges in consistently producing high-quality binding sites. Moreover, MIPs often perform less efficiently in complex, real-world samples compared to controlled laboratory environments, further restricting their widespread adoption [50]. The development of MIP-based sensors requires strong fundamental understanding of template–monomer–surface interactions. At present, most studies focus on creating potential prototypes rather than fully optimized commercial products. For example, the fabrication of QCM-MIP sensors remains costly and requires significant optimization steps, including careful evaluation of polymer growth conditions and analyte adsorption behavior.

In our experiments, geraniol removal was attempted using an acetonitrile–water cleaning mixture, which provides moderate solubility for hydrophobic compounds. However, QCM measurements indicated that significant adsorption remained (Δ*f* ≈ 10–38 Hz depending on exposure time), and EIS results further confirmed persistent surface blocking through elevated *R*_ct_ values. These findings indicate that geraniol interacts strongly with the gold surface and cannot be fully desorbed under the current cleaning conditions. Nevertheless, this adsorption behavior is important for understanding template removal challenges during imprinting. Experimental findings have shown that geraniol adsorbs strongly onto the sensor surface, preventing complete chemical cleaning and subsequently hindering pyrrole polymer adsorption. This limitation underscores the importance of surface preparation and material conservation, especially when working with expensive, single-use gold-coated sensors. Therefore, before advancing toward large-scale sensor development, further research into adsorption mechanisms and template extraction conditions must be prioritized to ensure reliable performance and cost-effectiveness. These aspects would influence the feasibility of imprinting in a future MIP system, enabling such sensors to move beyond prototypes and achieve practical commercialization in biosensing applications.

This study does not evaluate MIP performance but provides essential design criteria for the future fabrication of a functional geraniol-imprinted sensor. The QCM and EIS results reveal that geraniol exhibits strong and persistent adsorption to the gold surface, indicating that surface cleanliness and extraction solvent selection must be optimized to ensure accessible recognition cavities. Furthermore, the competitive adsorption observed between geraniol and pyrrole demonstrates the importance of carefully tuning the monomer-to-template ratio and polymer thickness to prevent surface blocking and ensure complete template inclusion within the polymer matrix. By applying these insights to controlled electropolymerization—adjusting the potential window, sweep rate, electrolyte composition, and layer formation (single vs. double layer)—the imprinting process in future experiments may be improved, providing better-defined interaction sites. These mechanistic findings therefore form the scientific basis for the next stage of research, where optimized parameters will be implemented to construct and test a selective MIP-based electrochemical sensor for geraniol detection.

Future research should aim at developing cheaper and greener synthesis methods. Work is needed to improve reproducibility of binding sites. Improved template extraction approaches for sensor surfaces must be created. MIPs should be tested more in complex, real-world samples. Sensor reusability aspects will need to be investigated when a functional MIP is developed. The cost of QCM-MIP sensors and gold substrates should be reduced. Combining MIPs with nanomaterials may improve sensitivity. Standardized protocols for synthesis and testing are also required.

## Figures and Tables

**Table 1:** Results of fitting EIS data according to the Randles equivalent circuit (inset in Figure 3). *CPE*_dl_—constant phase element of the electric double layer, α(*CPE*_dl_) is the dispersion coefficient, *R*_ct_ charge transfer resistance.

Solution	*CPE*_dl_, μF	α(*CPE*_dl_)	*R*_ct_, kΩ
1AD	14.02 ± 0.013	0.790 ± 0.001	2199.36 ± 114.82
1AD after CV	9.23 ± 0.039	0.852 ± 0.005	253.17 ± 4.40
2AD	7.15 ± 0.003	0.885 ± 0.001	8421.79 ± 3.82·10^−7^
2AD after CV	6.34 ± 0.012	0.878 ± 0.002	539.37 ± 6.64
1P	5.03 ± 0.003	0.822 ± 0.001	5441.26 ± 2.95·10^−6^
1P after CV	33.88 ± 0.130	0.663 ± 0.003	43.58 ± 0.36
2P	8.90 ± 0.015	0.797 ± 0.002	3991.70 ± 434.34
2P after CV	24.87 ± 0.050	0.698 ± 0.001	56.43 ± 0.24

**Table 2:** QCM Measurements of Pyrrole and Geraniol Adsorption.

Solution	Δf_av_ (Hz)	ΔR_av_ (Ohm)	t (min)	Solution	Δf_av_ (Hz)	ΔR_av_ (Ohm)	t (min)
A	−0.17	−0.32	[0–10]	A	−2.34	−4.03	[3–5]
B1	−11.24	−27.17	[20–23]	B2	−37	−26.74	[13–15]
A	−9.22	6.05	[37–39]	A	−8.84	−3.54	[20–22]
B1	−15.96	−25.06	[50–55]	B2	−52.57	−31.45	[33–35]
A	−23.39	8.18	[70–72]	A	−19.26	−9.36	[40–42]
B1	−29.38	−22.4	[75–95]	- -			
A	−26.47	13.3	[102–110]				

**Table 3:** Definitions of the two-electrode systems used in the experiment.

	System I—Classical Three-Electrode Setup	System II—QCM Sensor Setup
Electrodes	Working electrode: gold disk electrode (1 mm diameter). Reference electrode: Ag/AgCl (3 M KCl). Counter electrode: Pt disk electrode (2 mm diameter).	Electrode: 5 MHz gold-coated quartz crystal (1 mm diameter). Cell: custom-made from organic glass, stable against acetonitrile–water mixtures.
Solutions	Adsorption solutions: 1AD: 0.1 mol/L acetonitrile with LiClO_4_ (supporting electrolyte). 2AD: 0.005 mol/L geraniol in 1AD. Polymerization solutions: 1P: 0.05 mol/L pyrrole in 1AD. 2P: 0.05 mol/L pyrrole + 0.005 mol/L geraniol in 1AD.	Neutral solution A: water: acetonitrile (50:50 *v*/*v*), used as baseline. Solution B1: 0.05 mol/L geraniol in solution A. Solution B2: 0.5 mol/L pyrrole in solution A.
Techniques	CV, CA and EIS.	QCM
Techniques conditions	CV: In 1AD and 2AD: −500 mV to +1000 mV, 3 cycles, scan rate 100 mV/s, vs. Ag/AgCl. In 1P and 2P: −500 mV to +1000 mV, 5 cycles, scan rate 100 mV/s, vs. Ag/AgCl, max current 0.2 mA. EIS: frequency range 10,000 Hz → 2 Hz, 10 points per decade.	Flow-through QCM experiments at 1 mL/min, repeated twice.

**Figure A1:**
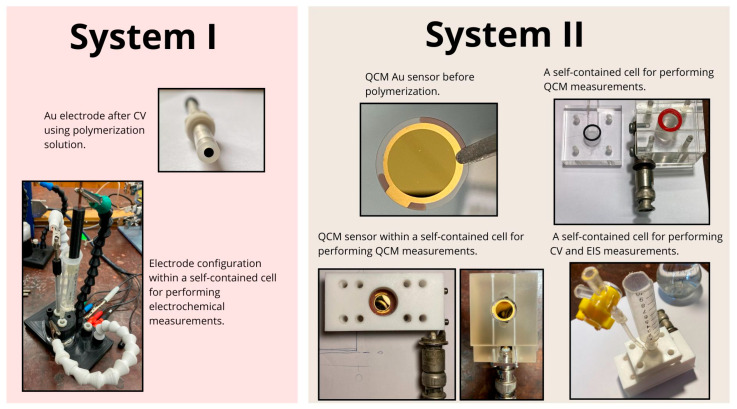
Systems I and II materials used. This figure shows the appearance of our handmade cell and electrodes used in Systems I and II. Both cells were custom-built and tested to ensure compatibility with our solutions, since acetonitrile can cause issues such as swelling, brittleness, and other forms of degradation in many cell materials.

## Data Availability

The data that supports the findings of this study are available from the corresponding author on reasonable request.

## Abbreviations

The following abbreviations are used in this manuscript:

Au	Gold
CA	Chronoamperometry
CV	Cyclic voltammetry
EO	Essential oil
EIS	Electrochemical impedance spectroscopy
HPLC	High-performance liquid chromatography
MIPs	Molecularly imprinted polymers
MIT	Molecular imprinting technology
Ppy	Polypyrrole
QCM	Quartz crystal microbalance
Qmax	Maximum adsorption capacity

## References

[1] Chen W., Viljoen A. (2010). Geraniol—A Review of a Commercially Important Fragrance Material. S. Afr. J. Bot..

[2] Mączka W., Wińska K., Grabarczyk M. (2020). One Hundred Faces of Geraniol. Molecules.

[3] Wróblewska A., Fajdek-Bieda A., Markowska-Szczupak A., Radkowska M. (2022). Preliminary Microbiological Tests of S-Carvone and Geraniol and Selected Derivatives of These Compounds That May Be Formed in the Processes of Isomerization and Oxidation. Molecules.

[4] Fahmy S.A., Nasr S., Ramzy A., Dawood A.S., Abdelnaser A., El-Said Azzazy H.M. (2023). Cytotoxic and Antioxidative Effects of *Geranium* Oil and Ascorbic Acid Coloaded in Niosomes against MCF-7 Breast Cancer Cells. ACS Omega.

[5] Sergazy S., Vetrova A., Orhan I.E., Senol Deniz F.S., Kahraman A., Zhang J.Y., Aljofan M. (2021). Antiproliferative and Cytotoxic Activity of Geraniaceae Plant Extracts Against Five Tumor Cell Lines. Future Sci. OA.

[6] Giongo J.L., de Almeida Vaucher R., Sagrillo M.R., Vianna Santos R.C., Duarte M.M.M.F., Rech V.C., Soares Lopes L.Q., Beatriz da Cruz I., Tatsch E., Moresco R.N. (2017). Anti-Inflammatory Effect of *Geranium* Nanoemulsion Macrophages Induced with Soluble Protein of Candida Albicans. Microb. Pathog..

[7] Malik M.N.H., Tahir M.N., Alsahli T.G., Tusher M.H., Alzarea S.I., Alsuwayt B., Jahan S., Gomaa H.A.M., Shaker M.E., Ali M. (2023). Geraniol Suppresses Oxidative Stress, Inflammation, and Interstitial Collagenase to Protect against Inflammatory Arthritis. ACS Omega.

[8] Ilić M., Samardžić S., Kotur-Stevuljević J., Ušjak D., Milenković M., Kovačević N., Drobac M. (2021). Polyphenol Rich Extracts of *Geranium* L. Species as Potential Natural Antioxidant and Antimicrobial Agents. Eur. Rev. Med. Pharmacol. Sci..

[9] Dumlupinar B., Karatoprak G.Ş., Demirci B., Akkol E.K., Sobarzo-Sánchez E. (2023). Antioxidant Activity and Chemical Composition of *Geranium* Oil and Its Synergistic Potential against Pneumococci with Various Antibiotic Combinations. Plants.

[10] El Azab E.F., Elguindy N.M., Yacout G.A., Elgamal D.A. (2020). Hepatoprotective Impact of Geraniol Against CCl4-Induced Liver Fibrosis in Rats. Pak. J. Biol. Sci..

[11] Ma J., Xu Y., Zhang M., Li Y. (2023). Geraniol Ameliorates Acute Liver Failure Induced by Lipopolysaccharide/D-Galactosamine via Regulating Macrophage Polarization and NLRP3 Inflammasome Activation by PPAR-γ Methylation Geraniol Alleviates Acute Liver Failure. Biochem. Pharmacol..

[12] Crespo R., Wei K., Rodenak-Kladniew B., Mercola M., Ruiz-Lozano P., Hurtado C. (2017). Effect of Geraniol on Rat Cardiomyocytes and Its Potential Use as a Cardioprotective Natural Compound. Life Sci..

[13] El-Bassossy H.M., Ghaleb H., Elberry A.A., Balamash K.S., Ghareib S.A., Azhar A., Banjar Z. (2017). Geraniol alleviates diabetic cardiac complications: Effect on cardiac ischemia and oxidative stress. Biomed. Pharmacother..

[14] Buch P., Sharma T., Airao V., Vaishnav D., Mani S., Rachamalla M., Gupta A.K., Upadhye V., Jha S.K., Jha N.K. (2023). Geraniol Protects Hippocampal CA1 Neurons and Improves Functional Outcomes in Global Model of Stroke in Rats. Chem. Biol. Drug Des..

[15] Bagheri S., Rashno M., Salehi I., Karimi S.A., Raoufi S., Komaki A. (2023). Geraniol improves passive avoidance memory and hippocampal synaptic plasticity deficits in a rat model of Alzheimer’s disease. Eur. J. Pharmacol..

[16] Chen W., Viljoen A.M. (2022). Geraniol—A Review Update. S. Afr. J. Bot..

[17] Azad T., Ahmed S. (2016). Common Milk Adulteration and Their Detection Techniques. Int. J. Food Contam..

[18] Nagraik R., Sharma A., Kumar D., Chawla P., Kumar A.P. (2021). Milk Adulterant Detection: Conventional and Biosensor Based Approaches: A Review. Sens. Bio Sens. Res..

[19] Zábrodská B., Vorlová L. (2015). Adulteration of Honey and Available Methods for Detection—A Review. Acta Vet. Brno.

[20] Pyell U., Pletsch-Viehmann B., Ramus U. (2002). Component Analysis of Vanilla Extracts and Vanilla Containing Commercial Preparations by Micellar Electrokinetic Chromatography or High-Performance Liquid Chromatography—A Method Comparison. J. Sep. Sci..

[21] Ávila M., Zougagh M., Escarpa A., Ríos Á. (2009). Fast Single Run of Vanilla Fingerprint Markers on Microfluidic-Electrochemistry Chip for Confirmation of Common Frauds. Electrophoresis.

[22] Capetti F., Marengo A., Cagliero C., Liberto E., Bicchi C., Rubiolo P., Sgorbini B. (2021). Adulteration of Essential Oils: A Multitask Issue for Quality Control. Three Case Studies: Lavandula Angustifolia Mill., *Citrus limon* (L.) Osbeck and Melaleuca Alternifolia (Maiden & Betche) Cheel. Molecules.

[23] Kalam S., Abu-Khamsin S.A., Kamal M.S., Patil S. (2021). Surfactant Adsorption Isotherms: A Review. ACS Omega.

[24] Yao C. (2000). Extended and Improved Langmuir Equation for Correlating Adsorption Equilibrium Data. Sep. Purif. Technol..

[25] Foo K.Y., Hameed B.H. (2010). Insights into the Modeling of Adsorption Isotherm Systems. Chem. Eng. J..

[26] Chen X., Ren Y., Qu G., Wang Z., Yang Y., Ning P. (2023). A Review of Environmental Functional Materials for Cyanide Removal by Adsorption and Catalysis. Inorg. Chem. Commun..

[27] Vasapollo G., Sole R.D., Mergola L., Lazzoi M.R., Scardino A., Scorrano S., Mele G. (2011). Molecularly Imprinted Polymers: Present and Future Prospective. Int. J. Mol. Sci..

[28] Kaspute G., Ramanavicius A., Prentice U. (2024). Molecular Imprinting Technology for Advanced Delivery of Essential Oils. Polymers.

[29] BelBruno J.J. (2019). Molecularly Imprinted Polymers. Chem. Rev..

[30] Parisi O.I., Francomano F., Dattilo M., Patitucci F., Prete S., Amone F., Puoci F. (2022). The Evolution of Molecular Recognition: From Antibodies to Molecularly Imprinted Polymers (MIPs) as Artificial Counterpart. J. Funct. Biomater..

[31] Önal Acet B., İnanan T., Salieva K., Borkoev B., Odabaşı M., Acet Ö. (2024). Molecular Imprinted Polymers: Important Advances in Biochemistry, Biomedical and Biotechnology. Polym. Bull..

[32] Guć M., Messyasz B., Schroeder G. (2021). Environmental Impact of Molecularly Imprinted Polymers Used as Analyte Sorbents in Mass Spectrometry. Sci. Total Environ..

[33] Coskun O. (2016). Separation Techniques: Chromatography. N. Clin. Istanb..

[34] Yin F., Xu F., Zhang K., Yuan M., Cao H., Ye T., Wu X., Xu F. (2021). Synthesis and Evaluation of Mesoporous Silica/Mesoporous Molecularly Imprinted Nanoparticles as Adsorbents for Detection and Selective Removal of Imidacloprid in Food Samples. Food Chem..

[35] Plausinaitis D., Sinkevicius L., Mikoliunaite L., Plausinaitiene V., Ramanaviciene A., Ramanavicius A. (2017). Electrochemical Polypyrrole Formation from Pyrrole ‘Adlayer’. Phys. Chem. Chem. Phys..

[36] Madadelahi M., Romero-Soto F.O., Kumar R., Tlaxcala U.B., Madou M.J. (2025). Electrochemical Sensors: Types, Applications, and the Novel Impacts of Vibration and Fluid Flow for Microfluidic Integration. Biosens. Bioelectron..

[37] Sharma P., Ghosh A., Tudu B., Bhuyan L.P., Tamuly P., Bhattacharyya N., Bandyopadhyay R., Das U. (2015). A Quartz Crystal Microbalance Sensor for Detection of Geraniol in Black Tea. IEEE Sens. J..

[38] Gangopadhyay D., Ankit, Naskar J., Kundu S., Nag S., Banerjee M.B., Roy R.B. Development of a Dual Sensor QCM Array Based Electronic Nose for Detection of Citronellal and Geraniol in Citronella Essential Oil. Proceedings of the 2024 IEEE Calcutta Conference (CALCON).

[39] Roy S., Nag S., Banerjee M.B., Dasgupta S., Pramanik P., Bandyopadhyay R. (2022). Detection of Geraniol in Palmarosa Essential Oil Using Silicone Sealant as Molecularly Imprinted Polymer in a QCM Sensor. Mater. NanoSci..

[40] Balciunas D., Plausinaitis D., Ratautaite V., Ramanaviciene A., Ramanavicius A. (2022). Towards Electrochemical Surface Plasmon Resonance Sensor Based on the Molecularly Imprinted Polypyrrole for Glyphosate Sensing. Talanta.

[41] Ratautaite V., Samukaite-Bubniene U., Plausinaitis D., Boguzaite R., Balciunas D., Ramanaviciene A., Neunert G., Ramanavicius A. (2021). Molecular Imprinting Technology for Determination of Uric Acid. Int. J. Mol. Sci..

[42] Marandi M., Kallip S., Sammelselg V., Tamm J. (2010). AFM Study of the Adsorption of Pyrrole and Formation of the Polypyrrole Film on Gold Surface. Electrochem. Commun..

[43] Sauerbrey G. (1959). Use of Quartz Crystal Vibrator for Weighting Thin Films on a Microbalance. Z. Phys..

[44] Brazys E., Ratautaite V., Mohsenzadeh E., Boguzaite R., Ramanaviciute A., Ramanavicius A. (2025). Formation of Molecularly Imprinted Polymers: Strategies Applied for the Removal of Protein Template (Review). Adv. Colloid. Interface Sci..

[45] Mohsenzadeh E., Ratautaite V., Brazys E., Ramanavicius S., Zukauskas S., Plausinaitis D., Ramanavicius A. (2024). Design of Molecularly Imprinted Polymers (MIP) Using Computational Methods: A Review of Strategies and Approaches. WIREs Comput. Mol. Sci..

[46] Plausinaitis D., Sinkevicius L., Samukaite-Bubniene U., Ratautaite V., Ramanavicius A. (2020). Evaluation of Electrochemical Quartz Crystal Microbalance Based Sensor Modified by Uric Acid-Imprinted Polypyrrole. Talanta.

[47] Ramanaviciene A., Ramanavicius A. (2004). Molecularly Imprinted Polypyrrole-Based Synthetic Receptor for Direct Detection of Bovine Leukemia Virus Glycoproteins. Biosens. Bioelectron..

[48] Ratautaite V., Topkaya S.N., Mikoliunaite L., Ozsoz M., Oztekin Y., Ramanaviciene A., Ramanavicius A. (2013). Molecularly Imprinted Polypyrrole for DNA Determination. Electroanalysis.

[49] Elgrishi N., Rountree K.J., McCarthy B.D., Rountree E.S., Eisenhart T.T., Dempsey J.L. (2018). A Practical Beginner’s Guide to Cyclic Voltammetry. J. Chem. Educ..

[50] Ashley J., Shahbazi M.-A., Kant K., Chidambara V.A., Wolff A., Bang D.D., Sun Y. (2017). Molecularly Imprinted Polymers for Sample Preparation and Biosensing in Food Analysis: Progress and Perspectives. Biosens. Bioelectron..

